# Natural phenolic derivatives based on piperine scaffold as potential antifungal agents

**DOI:** 10.1186/s13065-020-00676-4

**Published:** 2020-03-30

**Authors:** Jingjing Wang, Wenlong Wang, Haojin Xiong, Di Song, Xiufang Cao

**Affiliations:** College of Science, Huazhong Agricultural University, Wuhan, 430070 China

**Keywords:** Piperine, Essential oils, Synthesis, Fungicidal activity

## Abstract

Piperine is a natural alkaloid with a wide range of biological functions. Natural phenolic compounds existed in many essential oils (EOs) are plant-derived aroma compounds with broad range of biological activities, however, their actions are slow, and they are typically unstable to light or heat, difficult to extract and so on. In order to find high-potential fungicides derived from piperine, a series of piperine-directed essential oil derivatives were designed and synthesized. The structures of all molecules were confirmed by satisfied spectral data, including ^1^H NMR, ^13^C NMR and ESIMS. The target compounds were screened for their potential fungicidal activities against six species of plant pathogen fungi, including *Rhizoctonia solani*, *Fusarium graminearum*, *Phomopsis adianticola*, *Alternaria tenuis Nees*, *Phytophthora capsici* and *Gloeosporium theae*-*sinensis*. Some of target compounds exhibited moderate and broad-spectrum activity against tested fungi compared to the parental piperine. Further studies have shown that some different concentrations of compounds have significant inhibitory activity against *Alternaria tenuis Nees* and *Phytophthora capsici* compared to commercial carbendazim, and compound **2b** exhibited particularly significant broad-spectrum fungicidal activity.
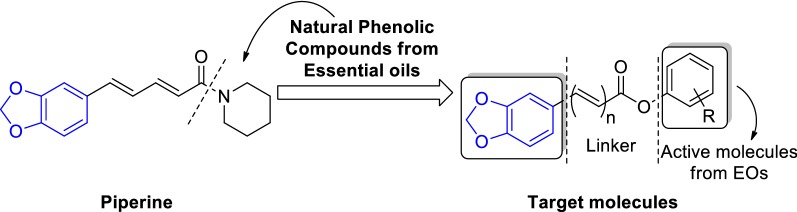

## Introduction

Piperine, a natural amide compound, is the main active substance extracted of *Piper nigrum Linn*. As an important natural alkaloid, piperine exhibited a wide spectrum of biological and pharmacological activities [1–6], it has anti-oxidation, antidepressant [7], toxic effect against hepatocytes [8], antiapoptotic efficacy [9], high immunomodulatory and antitumor activity [4], and has obvious effects in lowering blood fat [10]. Clinically, it can effectively control the incidence of hyperlipidemia, the treatment rate is as high as 93.3%, and it can also reduce the incidence of cardiovascular and cerebrovascular diseases. In addition to being used as a medicine, piperine is also an important organic synthetic building blocks and intermediate [11]. Its structure is mainly divided into three parts: piperidine ring, aromatic heterocyclic ring, and aliphatic hydrocarbon chain. These three places are usually considered by the researchers to be essential for their biological activity, and by modifying the structure of these parts, the biological activity of the compounds can be changed.

Essential oils (EOs) are class of complex mixtures of low molecular weight compounds extracted from various plants by steam distillation and various solvents [12]. Plant essential oils have received extensive attention from plant protection experts in recent years due to their low toxicity to mammals, low residue and extensive biological activity [13–15]. At present, there are many varieties of plant essential oils, and their applications are limited to the contact, fumigation and repellent of pests in confined environments such as greenhouses and warehouses [16–18]. In addition, essential oils can also be used as synergists, solubilizers, flavoring agents and chemical pesticides. However, most of essential oils are volatile, unstable to light and heat, easy to decompose, etc. Therefore, if the rational derivatization of essential oil molecules can be based on retaining their activity, the application of plant essential oils will undoubtedly be a significant development. Recently, during the course of our research for functional molecules based on natural essential oils [19, 20], a series of essential oil-oriented derivatives have been synthesized and approved to exhibit insecticidal or fungicidal activities, which suggest that these natural essential oils might contribute to the biological functions.

**Fig. 1:**
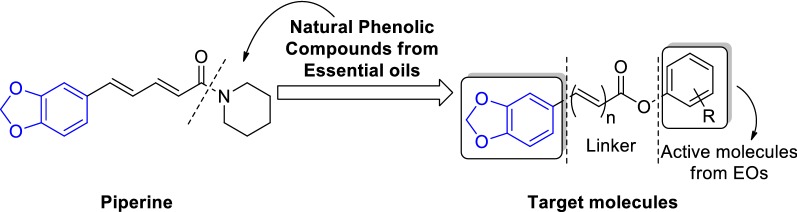
Design strategy of piperine-based essential oils derivatives

Based on this investigation, a series of piperine-oriented derivatives derived from natural phenolic compounds existed in essential oils were designed and synthesized as following strategy in Fig. 1. So, in order to explore the potential applications for these novel essential oil derivatives, we report herein the synthesis and characterization of twenty-one essential oil derivatives via simple reaction, and their antifungal activities against several phytopathogenic fungi have also been fully investigated.

## Materials and methods

### Instrumentation and chemicals

All chemicals or reagents used for syntheses were of analytical reagent, and used directly without purification. Melting points (m.p.) were determined on a RY-2 apparatus and are uncorrected. ^1^H NMR spectra were recorded on a Brucker spectrometer at 600 MHz with the CDCl_3_ as the solvent and TMS as the internal standard. ^13^C NMR spectra were recorded on a Brucker spectrometer at 150 MHz with CDCl_3_ as the solvent. Mass spectra were performed on a Waters ACQUITY UPLC^®^ H-CLASS PDA (Waters^®^) instrument. Column chromatography was carried out using silica gel 100–200 mesh. Analytical thin-layer chromatography (TLC) was carried out on precoated plates, and spots were visualized with ultraviolet light.

### General synthesis of precursors

The key precursors including (*E*)-3-(benzo[d][1,3]dioxol-5-yl)acrylic acid (n = 1) and piperic acid (n = 2) were prepared using a similar methods reported in the references [21, 22].

### General synthetic procedures for target compounds

The corresponding acid bearing 1,3-benzodioxole unit (0.005 mol), phenolic compound (0.005 mol) and acetonitrile (30–60 mL) were added to a 150 mL dry round bottom flask, and 0.3 g of 4-dimethylaminopyridine was added as a catalyst, and 1.5 g of *N*,*N*′-dicyclohexylcarbodiimide was further added as a condensing agent. The reaction was stirred at room temperature to 40 °C for additional hours, and TLC traced the reaction to completion. After the completion of the reaction, the solution was dissolved in water (20 mL), and the aqueous solution was extracted with ethyl acetate (30 mL × 2) twice. The combined organic phases were washed with 5% Na_2_CO_3_ solution (30 mL × 2) and water to neutrality and dried over anhydrous Na_2_SO_4_. After filtration and concentration, the corresponding crude compound were obtained, which were purified by silica gel column-chromatography (ethyl acetate/petroleum ether) or recrystallization to give pure compounds.

#### 5-Isopropyl-2-methylphenyl benzo[d][1,3] dioxole-5-carboxylate (**1a**)

^1^H NMR (600 MHz, CDCl_3_): *δ* = 7.85 (dd, *J* = 8.2, 1.8 Hz, 1H), 7.64 (d, *J *= 1.8 Hz, 1H), 7.19 (d, *J* = 7.8 Hz, 1H), 7.05 (dd, *J* = 7.8, 1.8 Hz, 1H), 6.98 (d, *J* = 1.8 Hz, 1H), 6.92 (d, *J *= 8.2 Hz, 1H), 6.08 (s, 2H), 2.93–2.88 (m, 1H), 2.18 (s, 3H), 1.25 (d, *J* = 6.6 Hz, 6H); ^13^C NMR (150 MHz, CDCl_3_): *δ* = 164.39, 152.25, 149.59, 148.23, 148.04, 131.03, 127.51, 126.26, 124.26, 123.63, 120.04, 110.08, 108.31, 102.08, 33.74, 24.07, 15.98; MS (ESI) *m/z* 299.6 (M+H)^+^, calcd. for C_18_H_19_O_4_*m/z* = 299.1.

#### 2-Isopropyl-5-methylphenyl benzo[d][1,3]dioxole-5-carboxylate (**1b**)

^1^H NMR (600 MHz, CDCl_3_): *δ *= 7.84–7.82 (m, 1H), 7.62 (s, 1H), 7.26–7.20 (m, 1H), 7.05 (d, *J* = 7.4 Hz, 1H), 6.94–6.88 (m, 3H), 6.06 (s, 2H), 3.06–3.01 (m, 1H), 2.33 (s, 3H), 1.20 (d, *J* = 7.2 Hz, 6H); ^13^C NMR (150 MHz, CDCl_3_): *δ* = 164.72, 152.15, 148.15, 147.93, 137.17, 136.60, 127.10, 126.43, 126.12, 123.51, 122.90, 109.91, 108.20, 101.96, 27.29, 22.67, 20.85; MS (ESI) *m/z* 299.5 (M+H)^+^, calcd. for C_18_H_19_O_4_*m/z* = 299.1.

#### Benzo[d][1,3]dioxol-5-yl benzo[d][1,3]dioxole-5-carboxylate (**1c**)

^1^H NMR (600 MHz, CDCl_3_): *δ* = 7.80 (dd, J = 8.2, 1.8 Hz, 1H), 7.59 (d, J = 1.8 Hz, 1H), 6.90 (d, J = 8.2 Hz, 1H), 6.81 (d, J = 8.4 Hz, 1H), 6.71 (d, J = 2.4 Hz, 1H), 6.63 (dd, J = 8.4, 2.4 Hz, 1H), 6.08 (s, 2H), 6.00 (s, 2H); ^13^C NMR (150 MHz, CDCl_3_): *δ* = 164.82, 152.20, 147.90, 145.36, 126.19, 123.30, 114.06, 109.92, 108.15, 108.01, 103.92, 101.97, 101.71; MS (ESI) *m/z* 287.5 (M+H)^+^, calcd. for C_15_H_11_O_6_*m/z* = 287.0.

#### 4-Allyl-2-methoxyphenyl benzo[d][1,3]dioxole-5-carboxylate (**1d**)

^1^H NMR (600 MHz, CDCl_3_): *δ* = 7.83 (dd, *J* = 8.2, 1.8 Hz, 1H), 7.62 (d, *J* = 1.8 Hz, 1H), 7.26 (s, 1H), 7.04 (d, *J* = 8.0 Hz, 1H), 6.89 (d, *J* = 8.2 Hz, 1H), 6.82 (d, *J* = 1.8 Hz, 1H), 6.06 (s, 2H), 6.00–5.95 (m, 1H), 5.16–5.06 (m, 2H), 3.80 (s, 3H), 3.40 (d, *J* = 7.2 Hz, 2H); ^13^C NMR (150 MHz, CDCl_3_): *δ* = 164.24, 152.02, 151.10, 147.80, 138.96, 138.21, 137.11, 126.27, 123.40, 122.66, 120.71, 116.12, 112.83, 110.12, 108.09, 101.88, 55.89, 40.12; MS (ESI) *m/z* 335.6 (M+Na)^+^, calcd. for C_18_H_16_NaO_5_*m/z* = 335.1.

#### 2,6-Dimethoxyphenyl benzo[d][1,3]dioxole-5-carboxylate (**1e**)

^1^H NMR (600 MHz, CDCl_3_): *δ* = 7.86 (dd, *J* = 8.2, 1.8 Hz, 1H), 7.66 (d, *J* = 1.8 Hz, 1H), 7.17–7.16 (m, 1H), 6.89 (d, *J* = 8.2 Hz, 1H), 6.64 (d, *J* = 8.4 Hz, 1H), 6.06 (s, 2H), 3.80 (s, 6H); ^13^C NMR ^1^H NMR (150 MHz, CDCl_3_): *δ* = 163.88, 152.57, 151.98, 147.76, 128.95, 126.41, 126.23, 123.32, 110.29, 108.07, 104.95, 101.85, 56.19; MS (ESI) *m/z* 325.5 (M+Na)^+^, calcd. for C_16_H_14_NaO_6_*m/z* = 325.1.

#### 2-Acetyl-5-methoxyphenyl benzo[d][1,3]dioxole-5-carboxylate (**1f**)

^1^H NMR (600 MHz, CDCl_3_): *δ* = 7.89 (d, *J* = 8.8 Hz, 1H), 7.84 (dd, *J* = 8.2, 1.8 Hz, 1H), 7.62 (d, *J* = 1.8 Hz, 1H), 6.92 (d, *J* = 8.2 Hz, 1H), 6.87 (dd, *J* = 8.8, 2.4 Hz, 1H), 6.70 (d, *J* = 2.4 Hz, 1H), 6.08 (s, 3H), 3.87 (s, 3H), 2.49 (s, 3H); ^13^C NMR (150 MHz, CDCl_3_): *δ* = 195.68, 164.42, 163.73, 152.39, 151.72, 147.98, 132.37, 126.49, 111.97, 110.07, 109.25, 108.30, 102.01, 55.75, 29.52; MS (ESI) *m/z* 337.4 (M+Na)^+^, calcd. for C_17_H_14_NaO_6_*m/z* = 337.1.

#### 2-(Methoxycarbonyl)phenyl benzo[d][1,3]dioxole-5-carboxylate (**1g**)

^1^H NMR (600 MHz, CDCl_3_): *δ* = 8.06 (dd, *J* = 7.8, 1.8 Hz, 1H), 7.85 (dd, *J* = 8.2, 1.8 Hz, 1H), 7.64 (d, *J* = 1.6 Hz, 1H), 7.61–7.59 (m, 1H), 7.36–7.34 (m, 1H), 7.22 (dd, *J* = 8.1, 0.8 Hz, 1H), 6.92 (d, *J* = 8.2 Hz, 1H), 6.08 (s, 2H), 3.76 (s, 3H); ^13^C NMR (150 MHz, CDCl_3_): *δ* = 165.04, 164.68, 152.19, 150.83, 147.89, 133.80, 131.87, 126.36, 126.00, 124.00, 123.48, 123.40, 110.07, 108.22, 101.94, 52.21; MS (ESI) *m/z* 323.4 (M+Na)^+^, calcd. for C_16_H_12_NaO_6_*m/z* = 323.1.

#### 5-Isopropyl-2-methylphenyl 3-(benzo[d][1,3]dioxol-5-yl)acrylate (**2a**)

^1^H NMR (600 MHz, CDCl_3_): *δ* = 7.79 (d, *J* = 16.2 Hz, 1H), 7.17 (d, *J* = 7.8 Hz, 1H), 7.11–7.02 (m, 3H), 6.93 (s, 1H), 6.03 (s, 2H), 6.84 (d, *J* = 8.0 Hz, 1H), 6.48 (d, *J* = 15.6 Hz, 2H), 6.03 (s, 2H), 2.91–2.87 (m, 1H), 1.24 (d, *J* = 7.2 Hz, 6H); ^13^C NMR (150 MHz, CDCl_3_): *δ* = 165.41, 149.94, 149.33, 148.03, 146.11, 128.66, 127.37, 124.85, 124.08, 119.84, 115.08, 106.60, 101.65, 33.58, 23.92, 15.86; MS (ESI) *m/z* 325.6 (M+H)^+^, calcd. for C_20_H_21_O_4_*m/z* = 325.1.

#### 2-Isopropyl-5-methylphenyl 3-(benzo[d][1,3]dioxol-5-yl)acrylate (**2b**)

^1^H NMR (600 MHz, CDCl_3_): δ = 7.78 (d, *J* = 16.2 Hz, 1H), 7.22 (d, *J* = 7.8 Hz, 1H), 7.13–7.01 (m, 4H), 6.87 (d, d, *J* = 7.8 Hz, 1H), 6.48 (d, *J* = 15.6 Hz, 1H), 6.03 (s, 2H), 3.05–3.01 (m, 1H), 2.33 (s, 3H) 1.21 (d, *J* = 7.2 Hz, 6H); ^13^C NMR (150 MHz, CDCl_3_): δ = 165.88, 149.95, 148.45, 147.99, 146.12, 137.21, 136.55, 128.65, 127.07, 124.87, 122.81, 115.16, 108.63, 106.63, 101.65, 27.16, 23.08, 20.86; MS (ESI) *m/z* 325.5 (M+H)^+^, calcd. for C_20_H_21_O_4_*m/z* = 325.1.

#### Benzo[d][1,3]dioxol-5-yl 3-(benzo[d][1,3]dioxol-5-yl)acrylate (**2c**)

^1^H NMR (600 MHz, CDCl_3_): *δ* = 7.68 (d, *J* = 15.6 Hz, 1H), 7.04–6.97 (m, 2H), 6.77 (d, *J* = 8.0 Hz, 1H), 6.73 (d, *J* = 8.4 Hz, 1H), 6.61 (d, *J* = 2.4 Hz, 1H), 6.53 (dd, *J* = 8.4, 2.4 Hz, 1H), 5.96 (s, 2H), 5.92 (s, 2H). 5.84 (s, 1H); ^13^C NMR (150 MHz, CDCl_3_): *δ* = 165.90, 150.02, 148.46, 148.00, 146.33, 145.29, 145.15, 128.60, 124.93, 114.97, 114.01, 108.65, 108.00, 106.60, 103.86, 101.69, 101.18; MS (ESI) *m/z* 313.5 (M+H)^+^, calcd. for C_17_H_13_O_6_*m/z* = 313.1.

#### 4-Allyl-2-methoxyphenyl 3-(benzo[d][1,3]dioxol-5-yl)acrylate (**2d**)

^1^H NMR (600 MHz, CDCl_3_): *δ* = 7.70 (d, *J* = 15.6 Hz, 1H), 7.05–6.92 (m, 3H), 6.79–6.69 (m, 3H), 6.42 (d, *J* = 15.9 Hz, 1H), 5.98–5.85 (m, 3H), 5.09–5.00 (m, 2H), 3.76 (s, 3H), 3.33 (d, *J* = 6.7 Hz, 2H); ^13^C NMR (150 MHz, CDCl_3_): *δ* = 165.32, 151.04, 149.87, 148.41, 146.15, 138.92, 138.03, 137.10, 128.76, 124.83, 122.67, 120.71, 116.15, 114.97, 112.76, 108.61, 106.65, 101.62, 55.90, 40.14; MS (ESI) *m/z* 361.6 (M+Na)^+^, calcd. for C_20_H_18_NaO_5_*m/z* = 361.1.

#### 2,6-Dimethoxyphenyl 3-(benzo[d][1,3]dioxol-5-yl)acrylate (**2e**)

^1^H NMR (600 MHz, CDCl_3_): *δ* = 7.72 (d, *J* = 15.6 Hz, 1H), 7.10–7.07 (m, 1H), 7.03 (d, *J* = 1.8 Hz, 1H), 7.00 (d, *J* = 7.5 Hz, 1H), 6.76 (d, *J* = 7.8 Hz, 1H), 6.57 (d, *J* = 9.0 Hz, 2H), 6.48 (d, *J* = 16.2 Hz, 1H), 5.96 (s, 2H), 3.76 (s, 6H); ^13^C NMR (150 MHz, CDCl_3_): *δ* = 164.89, 152.51, 149.82, 148.37, 146.23, 128.85, 126.22, 124.83, 114.83, 108.59, 106.69, 104.92, 101.60, 56.21; MS (ESI) *m/z* 351.5 (M+Na)^+^, calcd. for C_18_H_16_NaO_6_*m/z* = 351.1.

#### 2-Acetyl-5-methoxyphenyl 3-(benzo[d][1,3]dioxol-5-yl)acrylate (**2f**)

^1^H NMR (600 MHz, CDCl_3_): *δ* = 7.89 (d, *J* = 9.0 Hz, 1H), 7.82 (d, *J* = 15.6 Hz, 1H), 7.13 (d, *J* = 1.6 Hz, 1H), 7.11–7.09 (m, 1H), 6.87 (dd, *J* = 8.6, 2.4 Hz, 2H), 6.70 (s, 1H), 6.52 (d, *J* = 15.6 Hz, 1H), 6.06 (s, 2H), 3.88 (s, 3H), 2.54 (s, 3H); ^13^C NMR (150 MHz, CDCl_3_): *δ* = 195.96, 165.36, 163.69, 151.51, 150.14, 148.45, 147.07, 132.26, 128.51, 125.18, 123.68, 114.68, 111.89, 109.12, 108.64, 106.71, 101.69, 55.74, 29.61; MS (ESI) *m/z* 363.5 (M+Na)^+^, calcd. for C_19_H_16_NaO_6_*m/z* = 363.1.

#### Methyl 2-((3-(benzo[d][1,3]dioxol-5-yl)acryloyl)oxy)benzoate (**2g**)

^1^H NMR (600 MHz, CDCl_3_): *δ* = 7.97 (d, *J* = 9.0 Hz, 1H), 7.73 (d, *J* = 15.6 Hz, 1H), 7.53–7.50 (m, 1H), 7.27 (d, *J* = 8.0 Hz, 1H), 7.19 (s, 2H), 7.11 (d, *J* = 7.8 Hz, 1H), 7.04–7.00 (m, 2H), 6.77 (d, *J* = 8.0 Hz, 1H), 6.45 (d, *J* = 15.6 Hz, 1H), 5.96 (s, 2H), 3.77 (s, 3H); ^13^C NMR (150 MHz, CDCl_3_): *δ* = 165.61, 165.14, 150.65, 149.99, 148.43, 146.55, 133.79, 131.77, 128.67, 125.94, 125.01, 123.91, 123.56, 114.87, 108.61, 106.70, 101.66, 52.25; MS (ESI) *m/z* 349.4 (M+Na)^+^, calcd. for C_18_H_14_NaO_6_*m/z* = 349.1.

#### 5-Isopropyl-2-methylphenyl 5-(benzo[d][1,3]dioxol-5-yl)penta-2,4-dienoate (**3a**)

^1^H NMR (600 MHz, CDCl_3_): *δ* = 7.83–7.79 (m, *J* = 15.2, 10.9 Hz, 1H), 7.09 (d, *J* = 7.8 Hz, 1H), 6.96–6.95 (m, 3H), 6.88–6.80 (m, 3H), 6.75–6.70 (m, 2H), 6.09 (d, *J* = 15.2 Hz, 1H), 5.93 (s, 2H), 2.83–2.79 (m, 1H), 2.08 (s, 3H), 1.16 (d, *J* = 14.6 Hz, 6H); ^13^C NMR (150 MHz, CDCl_3_): *δ* = 165.51, 149.31, 148.79, 148.35, 148.04, 146.69, 141.25, 130.88, 130.40, 127.42, 124.33, 124.11, 123.34, 119.88, 119.20, 108.63, 105.93, 101.50, 33.61, 24.08, 15.92; MS (ESI) *m/z* 373.5 (M+Na)^+^, calcd. for C_22_H_22_NaO_4_*m/z* = 373.2.

#### 2-Isopropyl-5-methylphenyl 5-(benzo[d][1,3]dioxol-5-yl)penta-2,4-dienoate (**3b**)

^1^H NMR (600 MHz, CDCl_3_): *δ *= 7.55–7.51 (m, 1H), 7.15 (d, *J* = 7.8 Hz, 1H), 6.96–6.95 (m, 2H), 6.87–6.86 (m, 2H), 6.79 (s, 1H), 6.74–6.71 (m, 2H), 6.09 (d, *J* = 15.0 Hz, 1H), 5.92 (s, 2H), 2.96–2.91 (m, 1H), 2.24 (s, 3H), 1.12 (d, *J* = 6.6 Hz, 6H); ^13^C NMR (150 MHz, CDCl_3_): *δ* = 166.02, 148.81, 148.36, 147.97, 146.71, 141.32, 137.22, 136.60, 130.40, 127.11, 126.46, 124.33, 123.36, 122.87, 119.27, 108.64, 105.93, 101.52, 27.17, 22.75, 20.94; MS (ESI) *m/z* 373.5 (M+Na)^+^, calcd. for C_22_H_22_NaO_4_*m/z* = 373.2.

#### Benzo[d][1,3]dioxol-5-yl 5-(benzo[d][1,3]dioxol-5-yl)penta-2,4-dienoate (**3c**)

^1^H NMR (600 MHz, CDCl_3_): *δ* = 7.57 (dd, *J* = 15.2, 11.0 Hz, 1H), 7.02 (d, *J* = 1.6 Hz, 1H), 6.94 (dd, *J* = 8.0, 1.6 Hz, 1H), 6.88 (d, *J* = 15.0 Hz, 1H), 6.81–6.77 (m, 3H), 6.66 (d, *J* = 2.4 Hz, 1H), 6.58 (dd, *J* = 8.4, 2.4 Hz, 1H), 6.09 (d, *J* = 15.2 Hz, 1H), 5.99 (d, *J* = 9.6 Hz, 4H); ^13^C NMR (150 MHz, CDCl_3_): *δ* = 165.84, 148.81, 148.34, 147.95, 146.76, 145.23, 145.15, 141.34, 130.37, 124.27, 123.27, 119.07, 113.99, 108.59, 107.96, 105.95, 103.86, 101.65, 101.45; MS (ESI) *m/z* 339.2 (M+H)^+^, calcd. for C_19_H_15_O_6_*m/z* = 339.1.

#### 4-Allyl-2-methoxyphenyl 5-(benzo[d][1,3]dioxol-5-yl)penta-2,4-dienoate (**3d**)

^1^H NMR (600 MHz, CDCl_3_): *δ* = 7.64–7.60 (m, 1H), 7.16–7.13 (m, 1H), 7.03 (d, *J* = 1.8 Hz, 1H), 6.94 (dd, *J* = 8.0, 1.8 Hz, 1H), 6.87 (d, *J* = 15.6 Hz, 1H), 6.81–6.78 (m, 2H), 6.63 (d, *J* = 8.4 Hz, 2H), 6.22 (d, *J* = 15.2 Hz, 1H), 6.00 (s, 2H), 3.82 (s, 6H); ^13^C NMR (150 MHz, CDCl_3_): *δ *=164.83, 152.51, 148.68, 148.31, 146.65, 140.92, 130.53, 128.80, 126.14, 124.54, 123.14, 119.03, 108.55, 105.96, 104.92, 101.42, 56.19; MS (ESI) *m/z* 365.4 (M+H)^+^, calcd. for C_22_H_21_O_5_*m/z* = 365.1.

#### 2,6-Dimethoxyphenyl 5-(benzo[d][1,3]dioxol-5-yl)penta-2,4-dienoate (**3e**)

^1^H NMR (600 MHz, CDCl_3_): *δ* = 7.64–7.60 (m, 1H), 7.16–7.13 (m, 1H), 7.03 (d, *J* = 1.8 Hz, 1H), 6.94 (dd, *J* = 8.0, 1.8 Hz, 1H), 6.87 (d, *J* = 15.6 Hz, 1H), 6.81–6.78 (m, 2H), 6.63 (d, *J* = 8.4 Hz, 2H), 6.22 (d, *J* = 15.2 Hz, 1H), 6.00 (s, 2H), 3.82 (s, 6H); ^13^C NMR (150 MHz, CDCl_3_): *δ *= 164.83, 152.51, 148.68, 148.31, 146.65, 140.92, 130.53, 128.80, 126.14, 124.54, 123.14, 119.03, 108.55, 105.96, 104.92, 101.42, 56.19; MS (ESI) *m/z* 377.4 (M+Na)^+^, calcd. for C_20_H_18_NaO_6_*m/z* = 377.1.

#### 2-Acetyl-5-methoxyphenyl 5-(benzo[d][1,3]dioxol-5-yl)penta-2,4-dienoate (**3f**)

^1^H NMR (600 MHz, CDCl_3_): *δ* = 7.86 (d, *J* = 8.8 Hz, 1H), 7.63 (dd, *J* = 15.2, 11.0 Hz, 1H), 7.03 (d, *J* = 1.6 Hz, 1H), 6.98–6.87 (m, 3H), 6.86–6.78 (m, 4H), 6.66 (d, *J* = 2.5 Hz, 1H), 6.18 (d, *J* = 15.2 Hz, 1H), 6.00 (s, 2H), 3.85 (s, 3H), 2.51 (s, 3H); ^13^C NMR (150 MHz, CDCl_3_): *δ* = 192.87, 165.23, 163.65, 151.54, 148.89, 148.36, 147.49, 141.78, 132.19, 130.34, 124.26, 123.71, 117.79, 111.86, 109.06, 108.60, 105.98, 101.47, 55.71, 29.69; MS (ESI) *m/z* 389.4 (M+Na)^+^, calcd. for C_21_H_18_NaO_6_*m/z* = 389.1.

#### Methyl 2-((5-(benzo[d][1,3]dioxol-5-yl)penta-2,4-dienoyl)oxy)benzoate (**3g**)

^1^H NMR (600 MHz, CDCl_3_): *δ* = 7.95 (dd, *J* = 7.8, 1.8 Hz, 1H), 7.59–7.46 (m, 2H), 7.27–7.21 (m, 1H), 7.08 (dd, *J* = 8.2, 0.8 Hz, 1H), 6.95 (d, *J* = 1.6 Hz, 1H), 6.87 (dd, *J* = 8.0, 1.6 Hz, 1H), 6.81 (d, *J* = 15.6 Hz, 1H), 6.76–6.68 (m, 2H), 6.12 (d, *J* = 15.2 Hz, 1H), 5.92 (s, 4H), 3.76 (s, 2H); ^13^C NMR (150 MHz, CDCl_3_): *δ* = 164.51, 164.12, 149.61, 147.76, 147.31, 145.94, 140.29, 132.71, 130.70, 129.39, 124.85, 123.40, 122.89, 122.53, 122.22, 118.00, 107.55, 104.93, 100.43, 51.20; MS (ESI) *m/z* 375.5 (M+Na)^+^, calcd. for C_20_H_16_NaO_6_*m/z* = 375.1.

### Biological assay

The in vitro fungicidal activities of the target compounds **1a**–**3g** against *Rhizoctonia solani*, *Fusarium graminearum*, *Phomopsis adianticola*, *Alternaria tenuis Nees*, *Phytophthora capsici* and *Gloeosporium theae*-*sinensis* were evaluated using mycelium growth rate test, and all the procedure for bioassay were according to the methods reported in literature [23].

## Results and discussion

### Synthesis

**Scheme 1:**
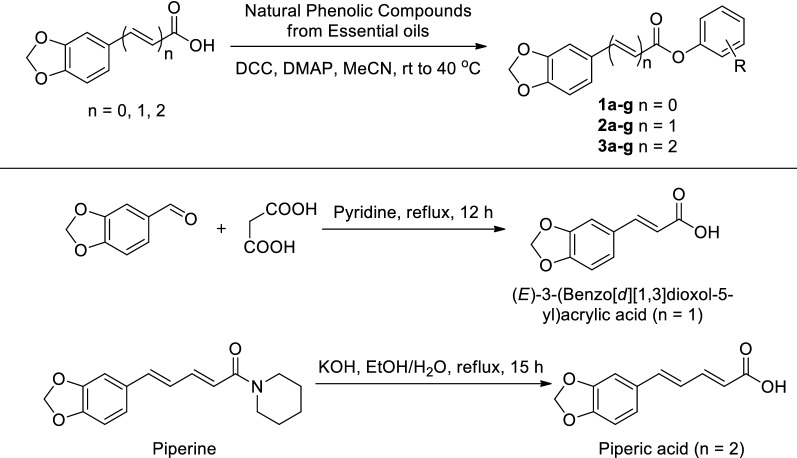
Synthetic route for intermediates and target molecules

A series of novel compounds **1a**–**g**, **2a**–**g** and **3a**–**g** derived from natural phenolic compounds existed in essential oils based on piperine scaffold can be synthesized by a mild and simple method as described in Scheme 1. In brief, the intermediate (*E*)-3-(benzo[*d*][1,3]dioxol-5-yl)acrylic acid (n = 1) can be prepared using piperonal as starting materials [21], and the other intermediate piperic acid (n = 2) was synthesized via basic hydrolysis reaction of piperine [22]. Then, all three acids were coupling with various essential oils molecules to obtain the corresponding esters using an optimization method.

To achieve the above goal for these essential oil derivatives, the initial experiment was optimized, and the different reaction conditions have been explored (Table 1). As can be seen from Table 1 (Entry 6 and 7), when the condensation system is EDCI/HOBT or CDI/DIPEA, TLC analysis showed that no obvious product was produced, however, the yields are improved when the condensation reactions are performed under the DCC/DMAP system. With this condition (DCC/DMAP) in hand, the solvent is further screened, and an equal volume of acetonitrile, tetrahydrofuran and dichloromethane are used as solvents. The reaction time and temperature are the same. The relationship between solvent and yield was obtained, as shown in Table 1, when acetonitrile was the solvent, the yield was the highest. In order to investigate the effect of the target compound yield on the reaction temperature, the experiment was carried out at a reaction temperature of 40 °C, 60 °C, and 90 °C, respectively. The results show that the yield gradually decreases with increasing temperature, and the yield is highest at 40 °C. Finally, we determined the optimal synthetic conditions for the synthesis of pepper acid-directed essential oil derivatives: DCC/DMAP is a catalytic condensation system, the solvent is acetonitrile, the reaction temperature is 40 °C, and the yield of the target compound is 83.40%.

**Table 1:** The optimal reaction conditions of piperic acid and carvacrol

Entry	Catalytic system	Solvent	Temperature (°C)	Time (h)	Yield (%)
1	DCC/DMAP	THF	40	6	47.47
2	DCC/DMAP	DCM	40	6	78.42
3	DCC/DMAP	MeCN	40	6	83.40
4	DCC/DMAP	MeCN	60	6	68.55
5	DCC/DMAP	MeCN	90	6	65.46
6	EDCI/HOBT	MeCN	40	6	NR
7	CDI/DIPEA	MeCN	40	6	NR

All of the new natural phenolic derivatives were synthesized according to the optimal conditions described above, and the structures of all the obtained compounds in this study were confirmed by satisfactory spectral analysis, including ^1^H NMR, ^13^C NMR, ESI–MS. The chemical formulas of all compounds were described in Table 2, and their chemical structures and basic physicochemical properties were summarized in “Materials and methods”.

**Table 2:**
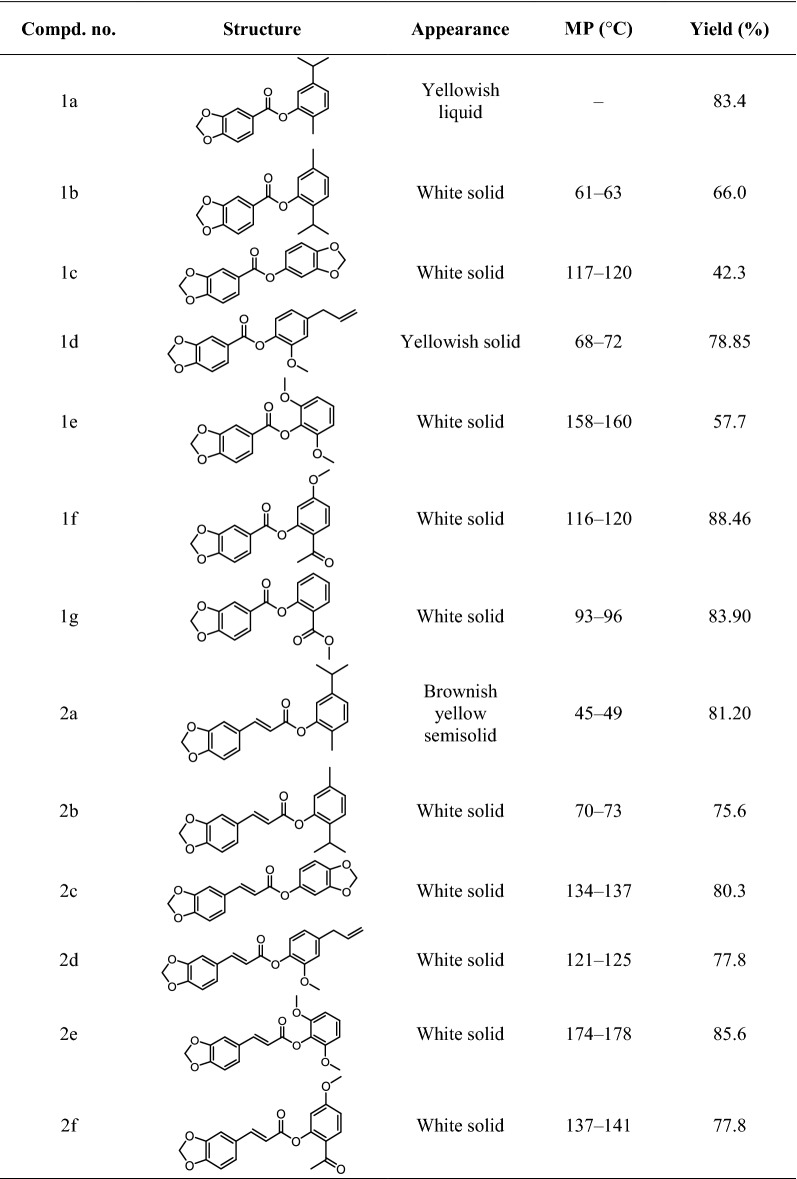

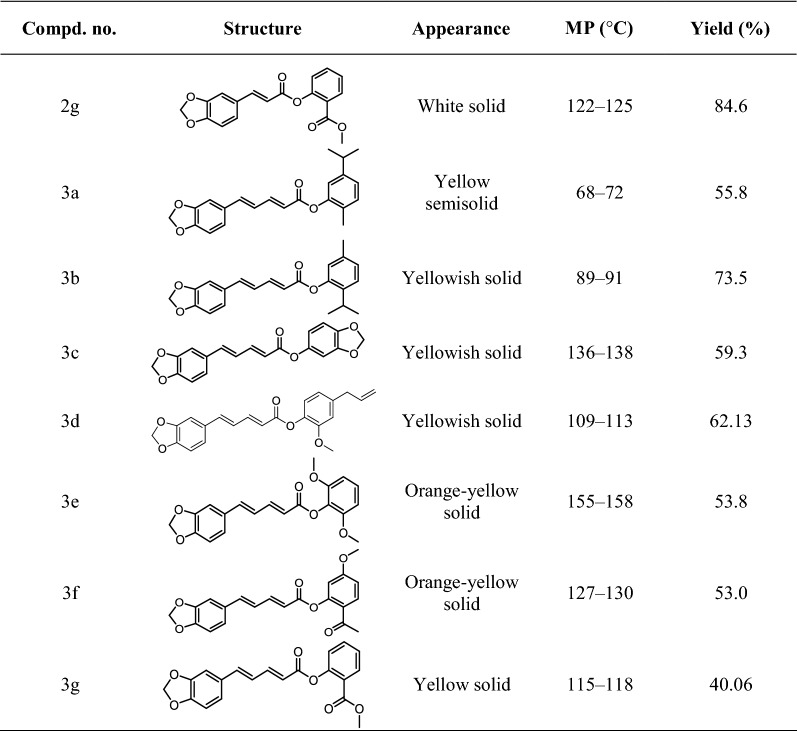
The chemical structure of target compounds **1a**–**3g**

### Spectrum analyses

**Fig. 2:**
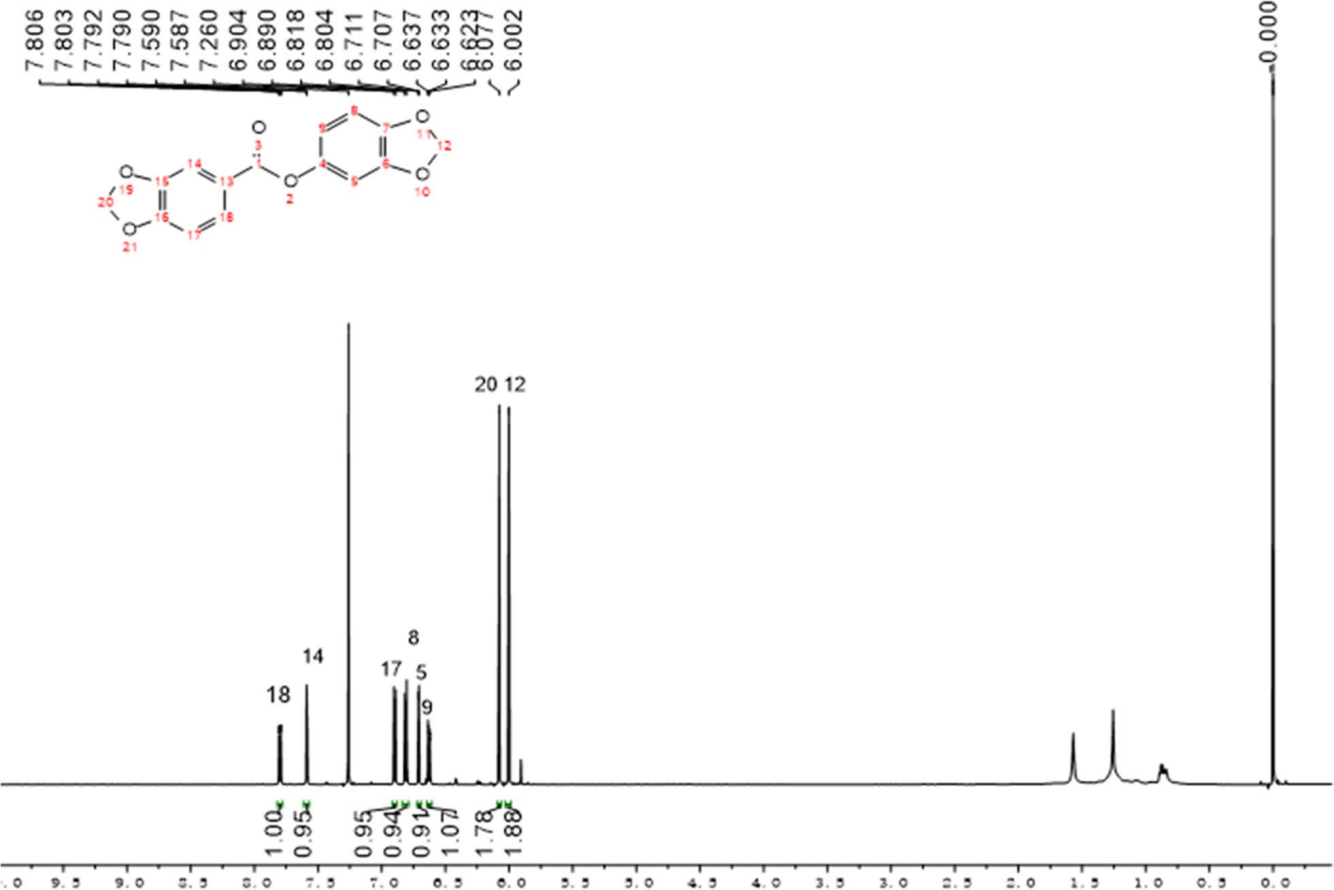
Representative ^1^H NMR spectra for compound **1c**

The structures of all target compounds **1a**–**3g** were confirmed by ^1^H NMR, ^13^C NMR (Additional file 1) and mass spectrometry, and their structures were well consistent with all the spectral data. A representative ^1^H NMR spectrum of **1c** is shown in Fig. 2, and each hydrogen shows a characteristic absorption peak. The methylene group on the piperine skeleton was not affected by other H in the ortho position, and a single peak appeared at 6.06 ppm, and the H of the benzene ring showed between 7.81 and 6.62 ppm.

### Biological activity

#### Primary screening test

In this study, all essential oil derivatives **1a**–**g**, **2a**–**g**, and **3a**–**g** were screened for their antifungal activities in *vitro* against six common plant pathogenic fungi (*Rhizoctonia solani*, *Fusarium graminearum*, *Alternaria tenuis Nees*, *Gloeosporium theae*-*sinensis*, *Phytophthora capsici*, *Phomopsis adianticola*), and the preliminary screening results were outlined in Table 3.

**Table 3:** In vitro fungicidal activity of target compounds **1a**–**3g**

Entry	Compd. no.	In vitro fungicidal activity (%)/100 µg/mL					
		*R.S*^a^	*F.G*^a^	*A.T*^a^	*G.T*^a^	*P.C*^a^	*P.A*^a^
1	**1a**	21.88	18.26	47.11	51.54	68.38	68.89
2	**1b**	29.69	36.52	52.89	35.77	31.62	61.85
3	**1c**	57.50	0.00	−1.65	4.62	14.53	3.70
4	**1d**	30.63	17.39	71.07	52.31	24.79	40.00
5	**1e**	17.50	15.65	25.62	27.69	29.06	3.70
6	**1f**	65.00	31.74	33.06	26.92	30.77	47.41
7	**1g**	20.94	22.61	7.44	0.00	47.86	23.70
8	**2a**	18.13	34.78	34.71	43.08	35.90	45.19
9	**2b**	57.50	62.61	52.07	66.92	100	100.00
10	**2c**	35.94	18.26	7.44	15.38	35.90	−3.70
11	**2d**	37.19	19.13	7.44	18.08	38.46	0.00
12	**2e**	63.75	21.74	−3.31	3.08	27.35	3.70
13	**2f**	40.94	20.87	2.48	13.85	33.33	−2.22
14	**2g**	46.56	11.30	9.92	60.77	47.01	29.63
15	**3a**	30.94	46.09	23.97	30.77	33.33	18.52
16	**3b**	25.00	35.65	25.62	15.38	23.08	16.30
17	**3c**	20.94	35.65	13.22	15.00	5.98	3.70
18	**3d**	21.25	45.22	24.79	38.46	19.66	17.04
19	**3e**	15.63	33.04	16.53	24.62	24.79	18.52
20	**3f**	7.81	37.09	16.53	23.08	0.85	29.63
21	**3g**	18.13	24.35	17.36	30.77	15.38	29.63
22	**Piperine**	63.13	53.04	66.12	76.92	41.88	29.63
23	**Carbendazim**	100.00	100.00	13.22	100.00	34.27	100.00

^a^R.S, *Rhizoctonia solani*; F.G - *Fusarium graminearum*, A.T, *Alternaria tenuis Nees*; G.T, *Gloeosporium theae-sinensis*; P.C, *Phytophthora capsici*; P.A, *Phomopsis adianticola*

Generally, as shown in Table 3, the preliminary assay illustrated that some compounds of the essential oil derivatives based on piperine displayed good inhibitory activities against some tested fungal strains, and we also can find that some of the target compounds have better inhibitory activities than piperine and carbendazim at the concentration of 100 µg/mL. Notably, six compounds displayed fungicidal activity more than 40% against *Rhizoctonia solani*, especially compound **1f** displayed an 65.00% inhibition rate, better than that of piperine (63.13%). Three compounds displayed fungicidal activity more than 40% against *Fusarium graminearum*, except compound **2b** displayed an 62.61% inhibition rate, better than that of piperine (53.04%). Four compounds displayed fungicidal activity more than 40% against *Alternaria tenuis Nees*, except compound **1d** displayed 71.07% inhibition rate, better than that of the piperine (66.12%) and carbendazim (13.22%). Five compounds displayed fungicidal activity more than 40% against *Gloeosporium theae*-*sinensis*, **2b** displayed an 66.92% inhibition rate, which is less than the activity of piperine (76.92%). Four compounds displayed fungicidal activity more than 40% against *Phytophthora capsici*, except compound **2b** displayed an 100% inhibition rate, which is much greater than the piperine (41.88%) and carbendazim (34.27%). Four compounds displayed fungicidal activity more than 40% against *Phomopsis adianticola*, except compound **2b** displayed an 100% inhibition rate, far superior to the piperine (29.63%).

#### Secondary screening test

The preliminary assay indicated many of the target compounds exhibited good fungicidal activities compared to the commercial fungicide carbendazim, in order to further investigate the potential fungicidal activities, we thus selected some compounds like **1a**, **1b**, **1c**, **1d**, **1g**, **2a**, **2b**, **2g** to have further exploration in such a situation, and compared the values of IC_50_ with piperine and carbendazim at different concentrations. The fungicidal activities expressed as IC_50_ values for highly potential compounds are listed in Table 4, which indicated some compounds had good inhibitory effects. As shown in Table 4, compounds **1a**, **1g**, **2b**, **2g** (IC_50_ = 11.21, 87.66, 7.79, 97.84 μg/mL) all displayed good inhibitory effects on *Phytophthora capsici* compared with the positive control carbendazim (IC_50_ > 100 μg/mL). Compounds **1a** and **2b** displayed good inhibitory effects compared with the piperine (IC_50_ = 34.87 μg/mL). In particular, **2b** exhibits a broad spectrum of bacteriostatic activity.

**Table 4:** The IC_50_ of some compounds against the plant pathogen fungi

Entry	Compd. no.	IC_50_^a^ (µg/mL)					
		*R.S*^b^	*F.G*	*A.T*	*G.T*	*P.C*	*P.A*
1	**1a**	39.92	156.99	43.06	64.65	11.21	35.67
2	**1b**	29.29	> 200	121.77	>200	–	45.75
3	**1c**	69.06	–	–	–	–	–
4	**1d**	–	–	–	142.36	–	–
5	**1g**	**–**	–	–	–	87.66	–
6	**2a**	89.50	–	–	–	–	72.66
7	**2b**	39.46	38.83	12.02	22.55	7.79	8.84
8	**2g**	**–**	–	>200	81.95	97.54	–
9	**Piperine**	89.50	>200	116.77	42.84	34.87	84.88
10	**Carbendazim**^c^	2.94	3.30	173.18	2.86	114.42	3.73

^a^IC_50_—compound concentration required to inhibit colony growth by 50%

^b^R.S, *Rhizoctonia solani*; F.G, *Fusarium graminearum*; A.T, *Alternaria tenuis Nees*; G.T, *Gloeosporium theae-sinensis*; P.C, *Phytophthora capsici*; P.A, *Phomopsis adianticola*

^c^Carbendazim, used as positive control

**Fig. 3:**
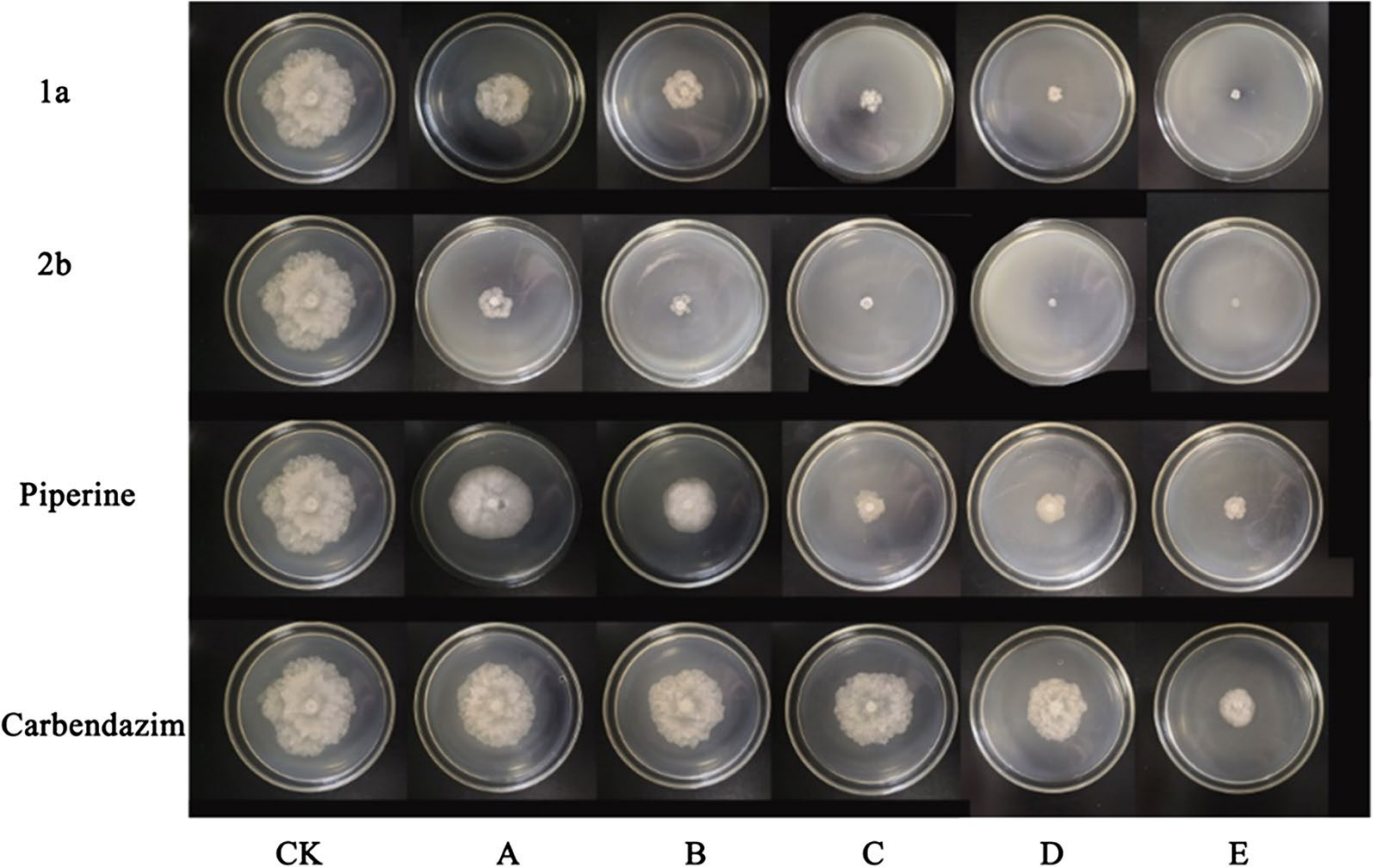
Inhibition activity of compounds **1a**, **2b**, piperine and carbendazim on *Phomopsis adianticola*. **a**–**e** The concentration of compounds 1a, 2b, piperine and carbendazim are 12.5, 25, 50, 100, and 200 µg/mL; *CK* blank control

In addition, the Fig. 3 indicated the inhibition effects of target compounds **1a**, **2b** on *Phomopsis adianticola* compared with that of piperine and carbendazim, which confirmed that the compounds **1a** and **2b** displayed the superior fungicidal activities on the *Phomopsis adianticola* at different concentrations of 12.5, 25, 50, 100, 200 µg/mL.

## Conclusions

In summary, 21 piperine-directed essential oil derivatives have been designed, synthesized and evaluated as potential fungicides. The structures of all obtained molecules were characterized by ^1^H-NMR, ^13^C-NMR and ESI–MS spectra analyses, and potential bioactivity was also assessed. Preliminary bioassay results indicate that some new compounds show better fungistatic activity than piperine. Among them, compound **2b** exhibits a broad spectrum of fungicidal activity, and it is hoped that further development of a new piperine-oriented agrochemicals.

## Supplementary information

**Additional file 1.**^1^H NMR, and ^13^C NMR spectra for the target compounds.

## Data Availability

All data generated or analyzed during this study are included in this published article.

## Abbreviations

EOs: Essential oils
m.p.: Melting points
TLC: Analytical thin-layer chromatography
R.S: *Rhizoctonia solani*
F.G: *Fusarium graminearum*
A.T: *Alternaria tenuis Nees*
G.T: *Gloeosporium theae-sinensis*
P.C: *Phytophthora capsici*
P.A: *Phomopsis adianticola*
