# Supplementary material for: Natural phenolic derivatives based on piperine scaffold as potential antifungal agents
Source: BMC Chem. 2020 Mar 30;14(1):24. doi: 10.1186/s13065-020-00676-4 (PMC7106752; doi:10.1186/s13065-020-00676-4)
Supplement: Supplementary file 1 — Additional file 1.1H NMR, and 13C NMR spectra for the target compounds. [file 13065_2020_676_MOESM1_ESM.docx]

1a

1b

1c

1d

1e

1f

1g

2a

2b

2c

2d

2e

2f

2g

3a

3b

3c

3d

3e

3f

3g
